# Comparison of post-treatment complications of conventional composites versus self-adhering composites: a review

**DOI:** 10.2340/biid.v13.45223

**Published:** 2026-03-25

**Authors:** Fatemeh Farshad, Erfan Eskandari, Marzieh Rohaninasab

**Affiliations:** a Dental Research Center, Dentistry Research Institute, Tehran University of Medical Sciences (TUMS), Tehran, Iran; b School of Dentistry, International Campus, Tehran University of Medical Sciences, Tehran, Iran; c Laser Research Center of Dentistry, Dentistry Research Institute, Tehran University of Medical Sciences, Tehran, Iran

**Keywords:** Composite resins, dental restoration repair, dental marginal adaptation, review, dental materials, postoperative complications

## Abstract

Self-adhering composites (SACs) are designed to simplify dental procedures by eliminating the need for separate adhesive systems. These materials are indicated for small Class I and Class V cavities, noncarious cervical lesions, and as liners in Class I and II restorations. Despite their advantages, the long-term durability and clinical performance of these composites compared to conventional composites (CCs) is a question. This study aims to compare post-treatment complications, including marginal adaptation, postoperative sensitivity, marginal discoloration, marginal gap, and microleakage, between CCs and SACs. In February 2024, a comprehensive search was conducted using PubMed, Google Scholar, Scopus, and Web of Science databases. No filters were applied during the search. Keywords included ‘resin composite restoration’, ‘post-operative sensitivity’, ‘marginal adaptation’, ‘marginal gap’, and ‘microleakage’. Studies were selected based on inclusion criteria (clinical trials, English-language, focusing on SACs vs. CCs) and exclusion criteria (nonindexed studies, conference abstracts, editorials, case reports, review articles, or pre-2019 studies). From 361 articles, 148 remained after removing duplicates and title screening. Out of 148 records found in databases, six records were selected for this study. Data showed that SACs and CCs have comparable marginal adaptation, postoperative sensitivity, marginal discoloration, and retention in conservative restorations. SACs showed slightly higher microleakage in some cases. Overall, the clinical performance of SACs were comparable to that of CCs. Thus, SACs can be a practical alternative for simplifying restorative procedures. However, their potential for higher microleakage needs further investigation through long-term clinical studies.

**KEY MESSAGES:**

- SACs exhibit comparable clinical performance to CCs in class V, I, and II restorations.
- Higher microleakage in some cases suggests that SACs may require further improvements in their formulation.

## Introduction

Resin composites play a significant role in modern restorative dentistry due to their esthetic properties and mechanical strength [1, 2]. These materials are typically light cured, so they are a suitable choice for direct restorations [1, 3, 4]. Despite their advantages, conventional composites (CCs) have some challenges, including polymerization shrinkage and sensitivity to thermal and chemical changes in the oral environment [2, 3, 5]. These factors can lead to complications such as marginal gaps, microleakage, postoperative sensitivity, and secondary caries [3, 5, 6].

The application of CCs involves a multistep bonding process, including acid etching, rinsing, drying, and adhesive application (primer and/or adhesive) [7, 8]. This process is technique sensitive. So, it needs a skilled clinician and a controlled environment to reduce risks from blood, saliva, or moisture [7, 9]. Each additional step increases the potential for procedural errors. These errors can lead to clinical failures such as debonding or marginal leakage [7, 9, 10]. To solve these challenges, self-adhering composites (SACs) were developed as a next-generation restorative material. SACs, when compared to CC systems, have the advantage of reducing the application time and the number of steps [11]. In addition, they integrate adhesive and restorative properties to eliminate the need for separate bonding systems [12, 13].

SACs are indicated for small Class I and Class V cavities, noncarious cervical lesions, and as liners in Class I and II restorations [6, 13]. Their application reduces procedure steps, time, and technique sensitivity. As a result, they are a good choice for pediatric or behavioral patients [9, 13]. However, there are some concerns about their long-term durability, bond strength, and clinical performance compared to CCs, particularly in restorations with higher stresses [10].

The clinical success of restorative materials depends on their sealing durability with dental tissues [6, 9]. Marginal adaptation reflects the absence of gaps between the restoration and tooth structure [9, 14]. On the other hand, microleakage allows bacterial infiltration, increasing the risk of secondary caries and restoration failure [15]. In addition, postoperative sensitivity indicates patient discomfort due to thermal, mechanical, or chemical stimuli. This sensitivity is often linked to exposed dentinal tubules or inadequate bonding [9, 16]. Moreover, marginal discoloration and gaps can lead to esthetic and functional failures [15]. Although SACs aim to simplify the restorative process, their performance in these critical areas compared to CCs remains questionable [12, 17].

This review explores whether SAC offers fewer complications than CC in conservative restorations. Moreover, it focuses on marginal adaptation, postoperative sensitivity, marginal discoloration, marginal gaps, and microleakage.

## Methods

### Search strategy

A systematic search was conducted in February 2024 across the following databases: National Library of Medicine MEDLINE/PubMed, Google Scholar, Scopus, and Web of Science databases. To ensure inclusion of the most recent studies, the search targeted peer-reviewed articles published between 2019 and 2023. No filters were applied during the search regarding study design. The present article does not have a registered protocol.

The search used specific keywords: ‘resin composite restoration’, ‘post-operative sensitivity’, ‘marginal adaptation’, ‘marginal gap’, and ‘microleakage’. These terms were selected based on their clinical relevance and frequent usage in restorative dentistry literature. Boolean operators (AND, OR) and truncation symbols were applied to optimize search sensitivity and specificity. For example: (‘resin composite restoration’ OR ‘composite filling’) AND (‘post-operative sensitivity’ OR ‘postoperative pain’) AND (‘marginal adaptation’ OR ‘marginal integrity’) AND (‘marginal gap’ OR ‘gap formation’) AND (‘microleakage’).

### Inclusion criteria

- Laboratory studies and clinical trials comparing post-treatment complications (marginal adaptation, postoperative sensitivity, marginal discoloration, marginal gaps, or microleakage) of CCs and SACs.
- Studies published in English.
- Studies conducted between 2019 and 2023.

### Exclusion criteria

- Nonclinical trial studies (e.g. case reports, reviews, expert opinions) except laboratory studies
- Studies not indexed in PubMed, Google Scholar, Scopus, or Web of Science.
- Studies published before 2019
- Studies presented as conference abstracts or lacking a proper statistical population.

### Study selection

**Figure 1:**
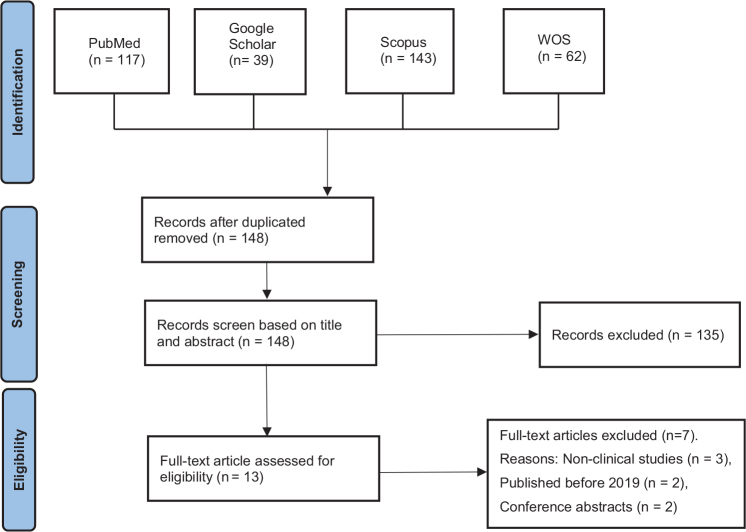
PRISMA-inspired flowchart of the number of studies identified, screened, eligible, and included in the evaluation.

Initially, the duplicate result was removed. The initial search had 361 articles. After removing 218 duplicates, 148 articles remained. Then, two independent reviewers (FF and EE) screened the articles at the title and abstract levels manually to choose studies comparing post-treatment complications of CCs and SACs. No attempt was made to blind the reviewers to the names of authors, institutions, and journals while making the assessment. Then, potentially eligible articles were independently assessed at the full-text level by FF, EE, and MR. Reviewer disagreements during both title/abstract and full-text screening stages were resolved through consensus discussions. In cases where consensus could not be reached between the two primary reviewers (FF and EE), a third reviewer (MR) was consulted to make the final decision. It should be mentioned that inter-rater reliability was not quantified using kappa statistics. The PRISMA-inspired flowchart is presented in Figure 1 to illustrate the study selection process.

### Study risk of bias assessment

Risk of bias analysis was done by two independent reviewers, previously calibrated. The Cochrane Risk of Bias for Randomized Trials version 2 (RoB 2) tool was used. This tool was structured into domains analyzing bias arising from the randomization process, deviations from intended interventions, missing outcome data, measurement of outcome, and selection of reported results [18]. For each domain, there are signaling questions that are designed to offer a systematic method for extracting information pertinent to assessing the risk of bias and offer answers: yes, probably yes, probably no, no, no information [19]. After responding to the signaling questions, the next step is to reach a risk-of-bias judgement, as follows: Low risk of bias, some concerns, or high risk of bias [18]. The RoB 2 tool incorporates algorithms that link responses to signaling questions to a suggested risk-of-bias assessment for each domain [18]. Any disagreement between the two assessors was solved by consulting a third assessor.

## Results

A total of 361 records were identified from the databases. After removing duplicates, 148 records remained. These articles were then screened by reviewing titles and abstracts. 135 studies that were either unrelated to the research focus, presented as abstracts, or lacked proper statistical data were removed. Thirteen full-text articles were assessed. Seven studies were excluded due to nonclinical study designs (*n* = 3), publication before 2019 (*n* = 2), or being conference abstracts (*n* = 2). Ultimately, six clinical studies were selected for detailed analysis based on their compliance with the inclusion criteria (Table 1).

**Table 1:** Summary of studies on the comparison of post-treatment complications of conventional composites versus self-adhering composites.

Ref	Type of study	Samples, caries risk	Composite	Results	Follow-up duration
[16]	Observational/*in vitro*	Box-only Class II/on the distal surface of 44 healthy human maxillary premolars/divided randomly into two groups (*n* = 22).	Group I – Gingival floor lined with Tetric N-Flow and was restored with Tetric N-Ceram; Group II – Gingival floor lined with Dyad flow and was restored with Herculite Precis.	There was no statistically significant difference between the study groups regarding the marginal adaptation.	None
[14]	Double-blind randomized, controlled clinical trial/ clinical evidence	Class I cavities/in 25 patients, with a total of 65 restorations/without uncontrolled rampant caries.	Group I – self-adhering flowable resin composite (VertiseFlow/Kerr-VR), Group II – flowable resin composite (Luxaflow/DMG-LX) in combination with an etch and rinse adhesive (Teco/DMG).	Similar mechanical and esthetic properties were reported for both materials.	Over a period of 1 to 5 years.
[17]	Randomized controlled trial/clinical evidence	Conservative Class I caries in 18 patients/participants randomly assigned to receive two pairs of restorations/without rampant caries.	Vertise™ flow Filtek™ Z350 XT Flowable.	The self-bonding composite has shown similar clinical performance to conventional flowable composite.	6 months.
[20]	Randomized clinical trial/clinical evidence	Cervical Class V caries/anterior teeth and premolars of 20 participants/ divided randomly into two groups (*n* = 10)/without deep carious defects.	Fusio Liquid Dentin (self-bonding flow composite) Tetric Evo Flow (conventional flow composite) was light cured using the total etch adhesive system.	Self-bonding composite and conventional flow composite have the same clinical performance in terms of hypersensitivity and marginal discoloration.	3, 6, and 12 months.
[21]	Randomized clinical split-mouth study/clinical evidence	Moderate Class II caries lesions/in 30 patients, each with two lesions.	Conventional bulk-fill composite (Filtek One, 3M Oral Care) self-adhesive bulk-fill restorative (SABF, 3M Oral Care).	It can be concluded from this study that both restorative materials received clinically acceptable scores in all FDI criteria examined.	6- and 12-month recalls
[22]	Observational/in vitro	Class V cavities/on 27 extracted premolars/divided randomly into three groups (*n* = 9).	Group 1: G-Aenial universal Flo with single bond (*n* = 9) (two-step etch and rinse system) Group 2: G-Aenial universal Flo with G-bond (*n* = 9) (single-step self-etch system) Group 3: Constic (*n* = 9) (self-bonding flow composite).	The difference in microleakage between the two stages of etch and rinse system self-bonding flowable composite (Constic) and the single stage of self-etch was statistically significant.	None

FDI: foreign direct investment.

These studies compared the clinical performance of SACs (e.g. Vertise Flow, Fusio Liquid Dentin, Constic, SABF) with CCs (e.g. Tetric N-Flow, Filtek Z350 XT Flowable, Luxa Flow, FOBF) in conservative restorations, focusing on marginal adaptation, postoperative sensitivity, marginal discoloration, marginal gaps, microleakage, retention, color match, and surface (Table 2).

**Table 2:** Comparative outcome chart of self-adhesive composite versus conventional composite.

Parameter	SACs	CCs	Comparison Outcome
**Marginal Adaptation**	Comparable, adequate sealing	Comparable	Both perform similarly
**Postoperative Sensitivity**	Minimal sensitivity, preserves smear layer	Minimal, occasional sensitivity	No significant difference
**Marginal Discoloration**	Similar discoloration rates	Similar discoloration rates	Both maintain esthetics
**Marginal Gap/Microleakage**	Mixed results, some higher leakage	Generally lower or comparable	Context dependent; SAC variable
**Retention**	High retention, reliable	High retention	Both have strong retention
**Color Match and Surface Gloss**	Comparable, differences diminish over time	Comparable	Both provide good esthetics

SAC: self-adhesive composite; CC: conventional composite.

### Risk of bias in studies (Table 3)

Clinical randomized controlled trials [14, 17, 20, 21] generally showed standardized clinical outcome measures, with low risk for outcome measurement and selective reporting. The main recurring concerns were incomplete description of allocation concealment and small sample size (especially Elshinawy et al. [21]), so several randomized controlled trials’ domains carry ‘some concerns’. The study of Oz et al. [14] was double blind and had the strongest long-term follow-up (5-year results).

**Table 3:** Risk of bias (RoB 2.0) of studies.

	D1	D2	D3	D4	D5
[16]	Unclear	Low	Low	Low	Low
[14]	Low	Low	Some concern	Low	Low
[17]	Low	Some concern	Low	Low	Low
[20]	Low	Some concern	Some concern	Low	Low
[21]	Low	Some concern	Low	Low	Low
[22]	Unclear	Low	Low	Low	Low

D1: Bias arising from the randomization process.

D2: Bias due to deviations from intended interventions.

D3: Bias due to missing outcome data.

D4: Bias in the measurement of the outcome.

D5: Bias in the selection of the reported result.

In vitro studies [16, 22] were well standardized in procedures and used objective imaging but commonly failed to report randomization methods, sample size calculations, and explicit blinding of assessors. These studies were marked as having unclear risks primarily for sample allocation and sample-size reporting.

### Marginal adaptation

Marginal adaptation was comparable between SACs and CCs across studies. Oz et al. [14] conducted a 5-year clinical trial comparing Vertise Flow as an SAC and Luxa Flow as a CC in Class I cavities [14]. At 1 year, 41.9% of Vertise Flow and 37.5% of Luxa Flow restorations exhibited minor marginal discrepancies (rated clinically good). By year 5, 27.3% of Vertise Flow and 41.7% of Luxa Flow restorations were rated clinically very good, with no statistically significant differences (*P* > 0.05) [14]. Gayatri et al. evaluated marginal adaptation in Class II cavities using scanning electron microscopy (SEM) and found no significant differences between Dyad Flow, a SAC, and Tetric N-Flow, a CC. Both materials showed comparable marginal integrity at the cementoenamel junction [16].

### Postoperative sensitivity

Postoperative sensitivity was minimal for both SACs and CCs, with no significant differences in most studies. Shaalan et al. compared Vertise Flow and Filtek Z350 XT Flowable in Class I cavities over 6 months. There was no sensitivity in either group [17]. In addition, Elshinawy et al. [21] evaluated Fusio Liquid Dentin and Tetric Evo Flow in Class V restorations over 12 months. No sensitivity was observed for Fusio Liquid Dentin at any time point, while one Tetric Evo Flow restoration showed sensitivity at 12 months, potentially due to acid etching exposing dentinal tubules [21]. Moreover, there were no significant differences between self-adhesive bulk-fill restorative (SABF, 3M Oral Care) and a conventional bulk-fill composite (Filtek One, 3M Oral Care; FOBF) in Class II cavities after 12 months in the study of Cieplik et al. [20].

### Marginal discoloration

Marginal discoloration was similar between SACs and CCs across evaluation periods. Oz et al. reported that 16.1% of Vertise Flow and 9.7% of Luxa Flow restorations showed minor marginal discoloration at 1 year, with 63.7% and 75% rated clinically very good by year 5, respectively (*P* > 0.05) [14]. In addition, Elshinawy et al. found no significant differences in marginal discoloration between Fusio Liquid Dentin and Tetric Evo Flow in Class V restorations over 12 months, attributing discoloration to high restoration stresses [21]. Finally, minimal marginal discoloration has been found in self-adhesive bulk-fill restorative (SABF, 3M Oral Care) restorations after 12 months in Cieplik et al., with no significant difference compared to conventional bulk-fill composite (Filtek One, 3M Oral Care; FOBF) [19].

### Marginal gap and microleakage

Microleakage and marginal gaps showed mixed results. Sengar et al. evaluated microleakage in Class V cavities using confocal laser microscopy. They compared Constic as an SAC with G-Aenial Universal Flo using etch-and-rinse (ER-2) or self-etch (SE-1) systems [22]. Constic exhibited the highest microleakage (mean score: 65.15) compared to ER-2 (46.65) and SE-1 (51.69), with significant differences (*P* < 0.05). This increased microleakage in SACs may be attributed to their lower filler content, which can reduce mechanical properties, and higher water sorption, which can lead to hydrolytic degradation over time. The lack of a separate adhesive step in SACs may also result in less effective hybrid layer formation, particularly in areas with high moisture, such as gingival margins [22]. However, Gayatri et al. found comparable marginal gaps between Dyad Flow and Tetric N-Flow in Class II cavities. This finding suggests that SACs perform adequately as liners [16].

### Retention

Retention rates were high for both SACs and CCs. Oz et al. reported 100% retention for both Vertise Flow and Luxa Flow at 1 year. Cumulative failure rates of Vertise Flow were 15.3% and Luxa Flow were 7.6% by year 5, showing no significant difference [14]. In addition, no retention loss was observed in Vertise Flow or Filtek Z350 XT Flowable after 6 months [17]. Finally, Cieplik et al. reported no significant retention differences between SABF and FOBF in Class II cavities after 12 months [20].

### Color match and surface

Color match and surface gloss were also comparable. Oz et al. reported that 9.7% of Vertise Flow and Luxa Flow restorations were rated clinically good for color match at 1 year [14]. Moreover, Elshinawy et al. found no significant differences in color match between Fusio Liquid Dentin and Tetric Evo Flow [21]. Although Cieplik et al. noted that conventional bulk-fill composite (Filtek One, 3M Oral Care; FOBF) showed slightly better surface gloss and color match initially, the differences reduce over time after polishing [20].

## Discussion

The findings of the six included studies indicate that SACs offer clinical performance comparable to CCs in conservative restorations, particularly for marginal adaptation, postoperative sensitivity, marginal discoloration, and retention [14, 17, 20, 21]. In line with our findings, a study conducted on posterior teeth compared the clinical performance of self-adhesive flowable resin with conventional flowable resins, showing comparable results to CC resins for the outcomes studied (anatomical form, secondary caries, marginal adaptation, color match, smoothness, retention, sensibility, and marginal staining) [11]. Moreover, SACs direct restorations demonstrate clinical performance similar to CCs or bulk-fill resin composites across all cavity types over a follow-up period of 6 to 48 months [18]. In addition, in comparison to resin-based sealant, the retention and marginal integrity of the self-adhering flowable composite were significantly higher [23]. These results suggest that SACs maintain reliable retention in conservative restorations [9, 13, 15]. Their simplified application, which eliminates acid etching and separate adhesive steps, reduces procedure time and technique sensitivity. This highlights SACs as a good choice for clinical settings where efficiency is critical, such as in pediatric dentistry or with uncooperative patients [9, 13]. The absence of acid etching in SACs may contribute to reduced postoperative sensitivity by preserving the smear layer [21]. This is particularly beneficial in deep cavities where we have higher dentinal tubule exposure [9, 16]. However, SACs exhibited higher microleakage in some studies, likely due to their flowable nature, lower filler content, and higher water sorption, which can weaken bond strength over time [22].

The chemical bonding mechanism of SACs involves functional monomers like glycerol phosphate dimethacrylate (GPDM) or 10-methacryloyloxydecyl dihydrogen phosphate (MDP). These monomers facilitate adhesion but may not achieve the robust micromechanical interlocking seen in CCs with ER-2or SE-1 adhesives [10, 12, 13]. These acidic monomers have a higher tendency to dissociate and degrade. They are likely to lend long-term instability to the chemical composition of adhesives. The presence of acidic monomers in SACs could make their ester linkages more susceptible to degradation over time [24].

SACs do not contain any water or solvents, which means that thermal degradation will not provoke hydrolysis phenomena. For both types of composites, however, the filler absorbance region showed higher values with aging. This may be related to thermal degradation of the silane coating of the filler particles, which consequently would expose the fillers [24].

Studies like Gayatri et al. and Alhumaid et al. suggest that SACs perform well as liners in low-stress restorations. Their flowability enhances cavity adaptation in these situations [15, 16]. However, their performance in high-stress areas, such as Class II restorations, may be limited due to increased polymerization shrinkage [6]. Moreover, some findings suggest that the SACs exhibited inferior shear bond strength compared with CCs when bonded with permanent dentin [25].

The flowable nature of SACs enhances their adaptability to cavity walls. Alhumaid et al. reported 90% marginal adaptation for Fusio Liquid Dentin as an SAC in Class V restorations, comparable to CCs [15]. Moreover, Bektas et al. (2013) reported similar sealing capabilities for SACs and CCs in enamel and dentin [6, 26]. These findings suggest that SACs achieve adequate marginal sealing, particularly in low-stress restorations [9, 12, 13]. However, this flowable nature of SACs may increase polymerization shrinkage, leading to marginal gaps or secondary caries in high-stress areas [3, 5, 6]. This limitation is particularly relevant for Class II restorations, where occlusal forces are higher [9]. As a result, their performance is context dependent [10]. The latest study, conducted in 2025, illustrated that the scores of microleakage in the gingival wall were lower in the CC after selective etching. However, there were no statistically significant differences in the scores of microleakage in the incisal wall. These findings emphasize that cavity location, depth, and substrate (enamel vs. dentin) significantly influence the performance of adhesive materials [11]. Despite this, SACs’ ease of use and reduced procedural steps make them a compelling alternative for conservative restorations [17, 20]. Their ability to achieve comparable clinical outcomes with fewer steps highlights their potential to improve clinical efficiency without compromising quality [12, 13].

Limitations of this review include the small number of included studies (six) and their relatively short follow-up periods (up to 5 years) [14, 17, 20, 21]. Understanding the differences in the effectiveness of the treatments can only be measured with greater accuracy over longer periods (≥10 years), as many clinical failures of restorations manifest late [11]. These may compromise the overall comprehensiveness of the result. Moreover, variability in SAC formulations, cavity types, and evaluation criteria across studies may also affect generalizability [9, 10]. For example, investigations focused on class V, I, and II restorations. Finally, the present article does not have a registered protocol. So, future research should focus on long-term clinical trials, standardized evaluation protocols, multicenter randomized controlled designs, extended follow-up periods, registration protocols, and diverse clinical scenarios to validate SAC performance and optimize their formulations for enhanced durability.

## Conclusion

SACs can offer a convenient alternative to CCs as they show similar clinical results in conservative class V, I, and II restorations while being simpler and less time consuming to apply. However, their tendency toward higher microleakage in some cases raises concerns about long-term durability. As a result, their reliability remains uncertain.

## Data Availability

Data sharing does not apply to this article, as no new data were created or analyzed in this study.
